# Feasibility of including patients with migration background in a structured heart failure management programme: A prospective case-control study exemplarily on Turkish migrants

**DOI:** 10.1371/journal.pone.0187358

**Published:** 2017-11-08

**Authors:** Roman Pfister, Peter Ihle, Birgit Mews, Elisabeth Kohnen, Marcus Wähner, Ute Karbach, Hasan Aslan, Hans-Wilhelm Höpp, Christian Alfons Schneider

**Affiliations:** 1 Department III of Internal Medicine, Heart Center, University of Cologne, Cologne, Germany; 2 HerzNetzKöln2, Cologne, Germany; 3 PMV Forschungsgruppe, University of Cologne, Cologne, Germany; 4 Institute of Medical Sociology, Health Services Research and Rehabilitation Science, Cologne, Germany; 5 Practice for general medicine, Cologne, Germany; 6 Kardiologie an der Pan Klinik, Cologne, Germany; Public Library of Science, UNITED STATES

## Abstract

**Aims:**

Structured management programmes deliver optimized care in heart failure patients and improve outcome. We examined the feasibility of including patients with migration background speaking little or no German in a heart failure management programme.

**Methods and results:**

After adaption of script material and staff to Turkish language we aimed to recruit 300 Turkish and 300 German (control group) patients within 18 months using the operational basis of a local heart failure management programme for screening, contact and inclusion.

Of 488 and 1,055 eligible Turkish and German patients identified through screening, 165 Turkish (34%) and 335 German (32%) patients consented on participation (p = 0.46). General practitioners contributed significantly more of the Turkish (84%) than of the German patients (16%, p<0.001). Contact attempts by programme staff were significantly less successful in Turkish (52%) than in German patients (60%, p = 0.005) due to significantly higher rate of missing phone numbers (36% vs 25%), invalid address data (28% vs 7%) and being unreachable by phone more frequently (39% vs 26%, all p<0.001). Consent rate was significantly higher in successfully contacted Turkish (63%) compared to German patients (50%, p<0.001).

**Conclusion:**

The inclusion of Turkish minority patients into a heart failure management programme is feasible with higher consent rate than in Germans. However, effort is high due to inherent logistic adaptions and barriers in identification and contacting of patients.

**Trial registration:**

DRKS00007780

## Introduction

Heart failure is a major public health problem in Western countries. Approximately 1–2% of the adult population has heart failure and in the context of ageing populations the prevalence is projected to increase by 23% till the year 2030 [1]. Despite drug and device therapy developed recently mortality of heart failure is high with about 20% per year [2]. The high morbidity of heart failure patients substantially affects quality of life [3] and causes enormous health care costs through an excess in hospitalizations [2,4]. Heart failure is one of the most frequent primary diagnosis causing hospitalizations in adults resulting in enormous health care costs [5].

Structured multidisciplinary programmes have been shown to deliver optimized care in chronic heart failure patients, improve quality of life and reduce hospitalization rates and associated health care costs [6,7]. Current guidelines give a strong recommendation on the enrollment of heart failure patients in multidisciplinary-care management programmes [8]. Besides limitations in availability of such programmes a potential shortcoming in clinical reality is that not all patients will have access. For instance, there is evidence from the U.S. showing that ethnic minority patients have less access to advanced medical care and guideline based diagnostics and treatment, and show less therapy adherence and have less knowledge on their disease [9–12]. Since this might at least partly explain the adverse clinical outcome [13] observed in minority heart failure patients there is a particular need of multidisciplinary management programmes which address many of the above deficiencies in these patients.

In the European Union the rate of people with background of immigration is about 7%, with distinct countries such as UK and Germany reporting rates of 10 to 20%. So far data on potential barriers limiting the accession to heart failure management programmes particularly in the context of ethnic minorities are lacking. Aim of this study was to examine the feasibility of including Turkish patients which in Germany is the dominant nationality of immigrants, speaking little or no German in a regional nurse-led heart failure management programme and identify potential barriers [14].

## Material and methods

This observational, controlled study was an independent part of the project “Gender and migration associated barriers and outcomes in structured heart failure management programmes” (GemaB). The study complies with the Declaration of Helsinki, was approved by the ethics committee of the University hospital of Cologne (13–041) and all patients gave written informed consent for participation.

### The heart failure management programme

The regional heart failure management programme “Heart Network Cologne” (Herz Netz Köln, HNC) which was implemented in the region of Cologne, Germany, in 2007 was used as an operational basis for this study. HNC is a nurse-coordinated programme including key elements recommended in recent guidelines of the European Society of Cardiology (ESC) [8,15] such as trans-sectoral care of patients, education, adherence to treatment guidelines, facilitated access to specialist heart failure cardiologists and inpatient treatment and follow-up monitoring including home-based visits, telephone contact and tele-monitoring for assessment of unexplained weight gain, functional and psychosocial status and quality of life. HNC is supported by the two largest public health insurance companies (Barmer GEK and AOK) and patients eligible for inclusion had to live in the area of Cologne, a city with about 1 Mio citizens, be health insured by either of the two companies, be aged older than 14 years, speak German and have a doctor-based diagnosis of chronic heart failure according to the recommendations of the ESC with NYHA class II or higher [8]. For the purpose of this study, the inclusion criteria were extended to Turkish patients health insured by the two respective companies speaking little or no German.

In order to overcome the linguistic communication barrier of Turkish patients and potential requirements in cultural conformity of care providers, two additional heart failure nurses with Turkish immigration background and speaking Turkish were trained who were responsible for all contacts and care of Turkish patients. Also, all script material for administration, patient information and consent was translated into Turkish.

### Participating physicians

General physicians (GP), ambulatory cardiologists and cardiological hospital departments and clinics already collaborating with HNC for recruitment and treatment of patients were asked to participate in this study. Preferentially practices with high potential of Turkish heart failure patients identified from administrative data through ICD-10 code I50.x provided by the health insurance companies were contacted. In contrast to experiences with German patients who were essentially recruited by cardiologists, very few Turkish patients with diagnosis of heart failure were treated by participating cardiologists. Accordingly, additional GP practices so far not collaborating with HNC with high potential of Turkish heart failure patients were identified, contacted and asked for participation.

### Patient recruitment

**Fig 1:**
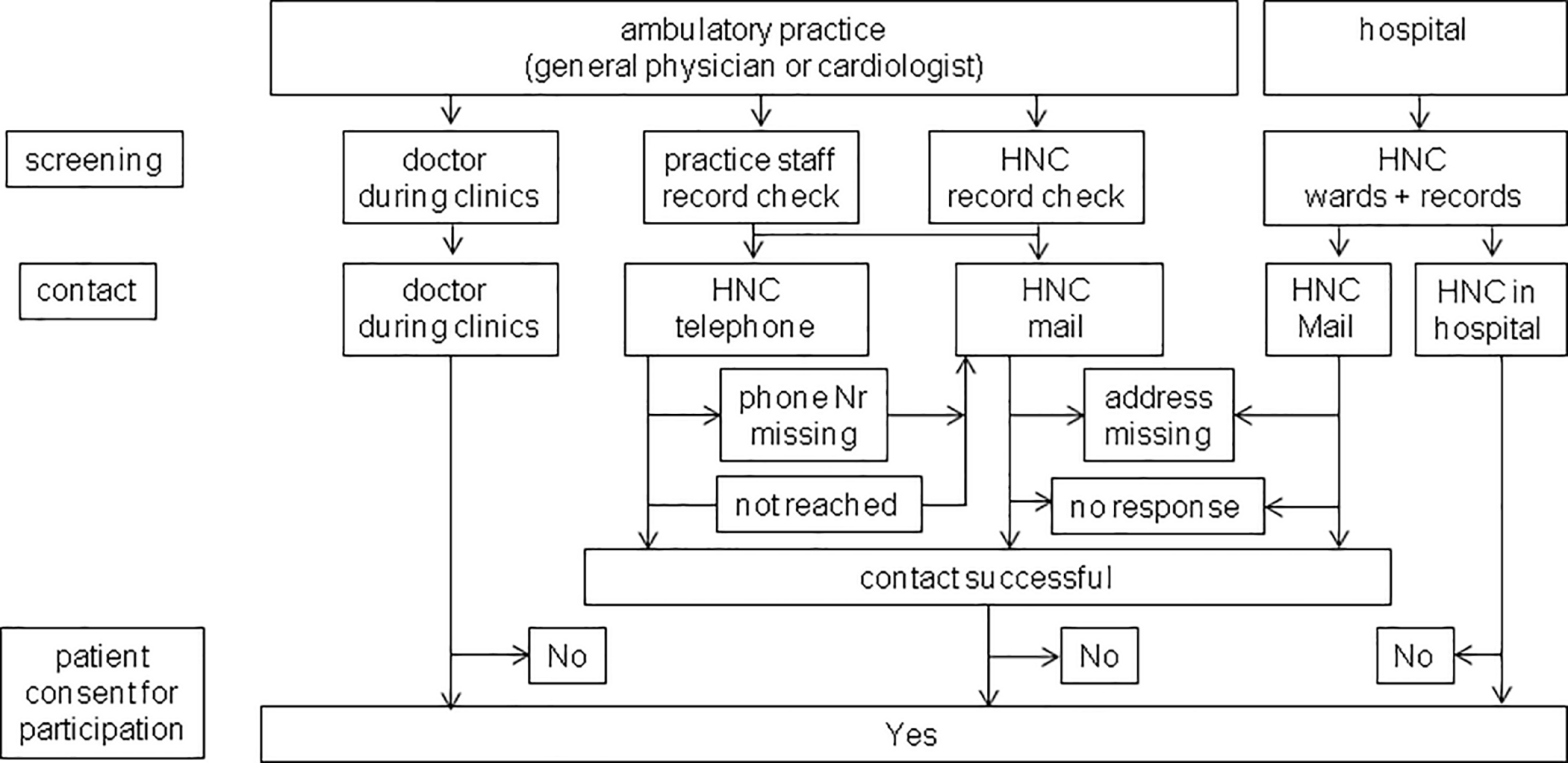
Pathways for screening and contacting patients for participation in the heart failure management programme. HNC indicates staff of the programme.

Screening for and contacting of potentially eligible patients was performed for ambulatory patients in doctor`s practices (GP or ambulatory cardiologist) and for inpatients in hospitals according to the requirements of the respective institutions and were not standardized (Fig 1). In practices screening was performed either by the physician during clinics, by the physician or practice assistants through record check or, if permitted by the physician, the preferred option was screening by staff of HNC through record check using diagnosis-code and drug-class based standardized search criteria (diagnosis of heart failure, coronary heart disease or prescription of diuretics). In the first case, the physician directly introduced the programme to the patient and asked for written informed consent for participation. In the latter two cases, after eligibility of screened patients was approved by the treating physician, patients were contacted by the HNC staff either by telephone call or by mail letter to make an appointment for an informational talk where heart failure nurses introduced the programme to the patient and asked for written informed consent for participation. The order of primary and secondary contact pathway (phone or mail) by HNC staff was also purported by the treating physician and differed across institutions. Telephone contact attempts by HNC staff in every patient followed a pre-specified protocol with several calls at different days of the week and different hours of the day including working and non-working hours.

In hospitals cardiological inpatients and patients attending the heart failure outpatient clinic were screened by physicians and HNC staff and, if eligibility was approved by the treating physician, patients were informed about the programme and asked for written informed consent for participation. Additionally, patient hospital records of the last year before start of the study were screened by HNC staff using the discharge code I50.x. Eligible patients were invited to an informational presentation of the programme and asked for written informed consent for participation.

Based on previous experience on the recruitment of German patients in HNC we aimed to include 300 patients in the German and in the Turkish group each within a period of 21 months.

### Statistics

Continuous variables are presented as median and interquartile range. Categorical variables are presented as frequency and rate. Comparisons across categories were performed using chi-2 test. All p-values are two-sided and <0.05 is considered statistically significant.

## Results and discussion

### Participating physicians and institutions

Fifteen of 97 (15%) GP practices and 11 of 11 (100%) cardiological practices which were contacted agreed to participate in the study. Eleven of the participating GP practices and 1 of the cardiological practices were no previous collaborators of HNC and were newly selected because of high potential of Turkish heart failure patients. Ten of the newly identified GP practices were led by Turkish physicians. Additionally, the cardiological department of a tertiary care hospital in Cologne was contacted and agreed to participate.

### Patient recruitment

**Fig 2:**
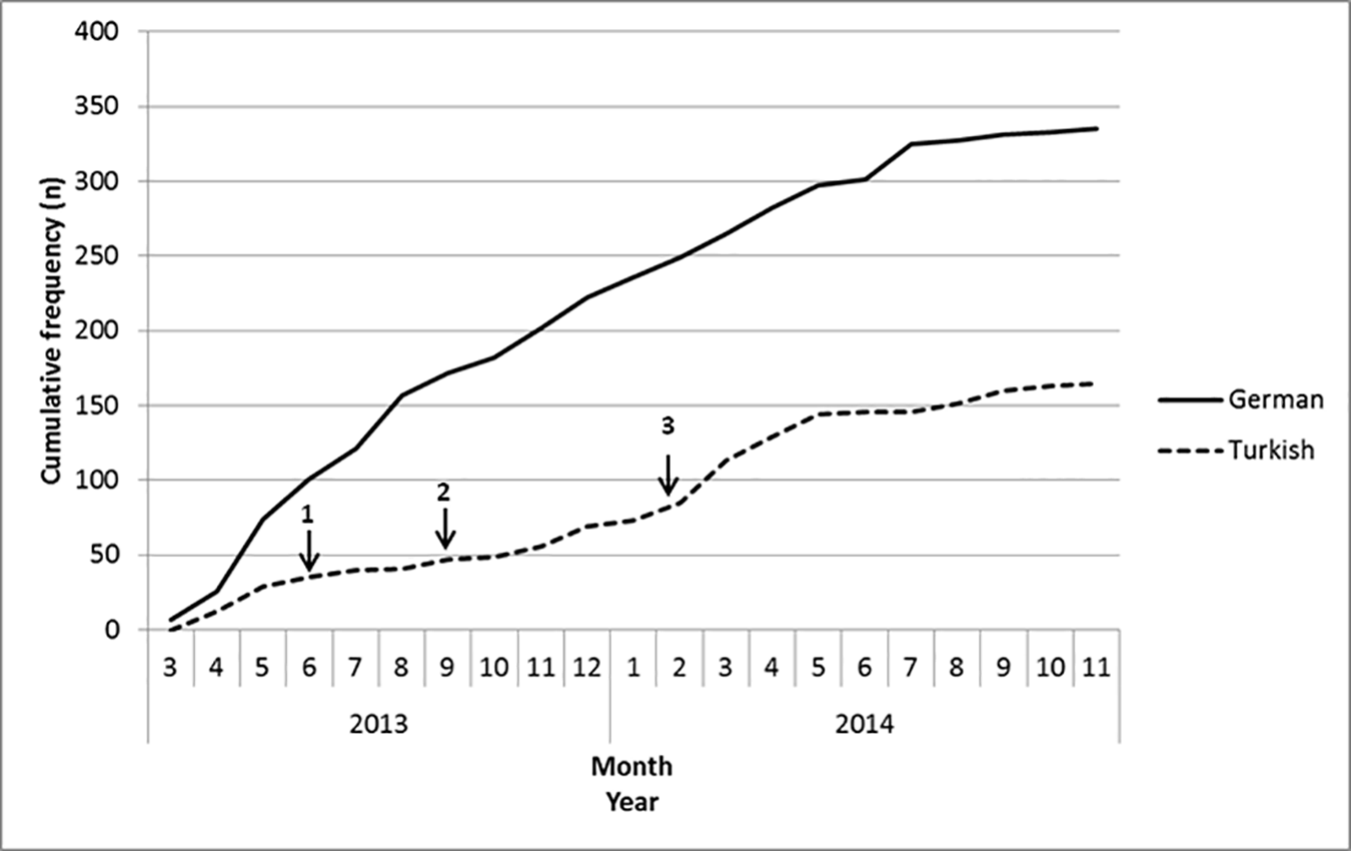
Cumulative frequency of German and Turkish consented participants over the recruitment period. Arrows and numbers indicate initiation of actions taken to increase recruitment of Turkish patients (1 and 2: programme promotion in Turkish culture and health care institutions, 3: opening to other health care insurances).

Recruitment of German patients developed as expected and finally 335 German patients (42% women, median [interquartile range] age 73, 64–79 years) consented on participation in the programme (Fig 2). Recruitment of Turkish patients was lower than expected and finally 165 Turkish patients consented on participation (48% women [p = 0.25 for difference to German patients], median [interquartile range] age 69, 63–73 years [p<0.001 for difference to German patients]). German participants showed more severe stages of heart failure than Turkish participants with a significantly higher NYHA class (class III/IV 51% vs 37.5%, p = 0.03) and a significantly higher rate of heart failure with reduced ejection fraction (24.3% vs 12.7%, p = 0.04). Turkish and German patients did not differ significantly regarding blood pressure, heart rate and rate of ischemic origin of heart failure (all p>0.2).

Because of the low recruitment rate actions to increase recruitment of Turkish patients were initiated (S1 Table). Providing information material and presentations within highly frequented institutions of the Turkish culture area and health care institutions was not effective (Fig 2). Integrating another cardiological hospital department with high rate of Turkish patients in the recruitment process and free of cost disposition of BNP point-of-care assays in 2 Turkish GP practices to motivate identification of potential heart failure patients and initiate further cardiological check-up also did not increase recruitment. The only action which increased recruitment frequency of Turkish patients was the opening of the programme to patients with other health care insurance companies than those two defined in inclusion criteria (Barmer GEK and AOK). In the final sample of consented participants 8% of German and 41% of Turkish patients were insured by other than the two initially accepted health care companies, with 30% of Turkish patients being insured by a company (BKK pronova) associated with a large local employer with high rate of Turkish employees.

**Fig 3:**
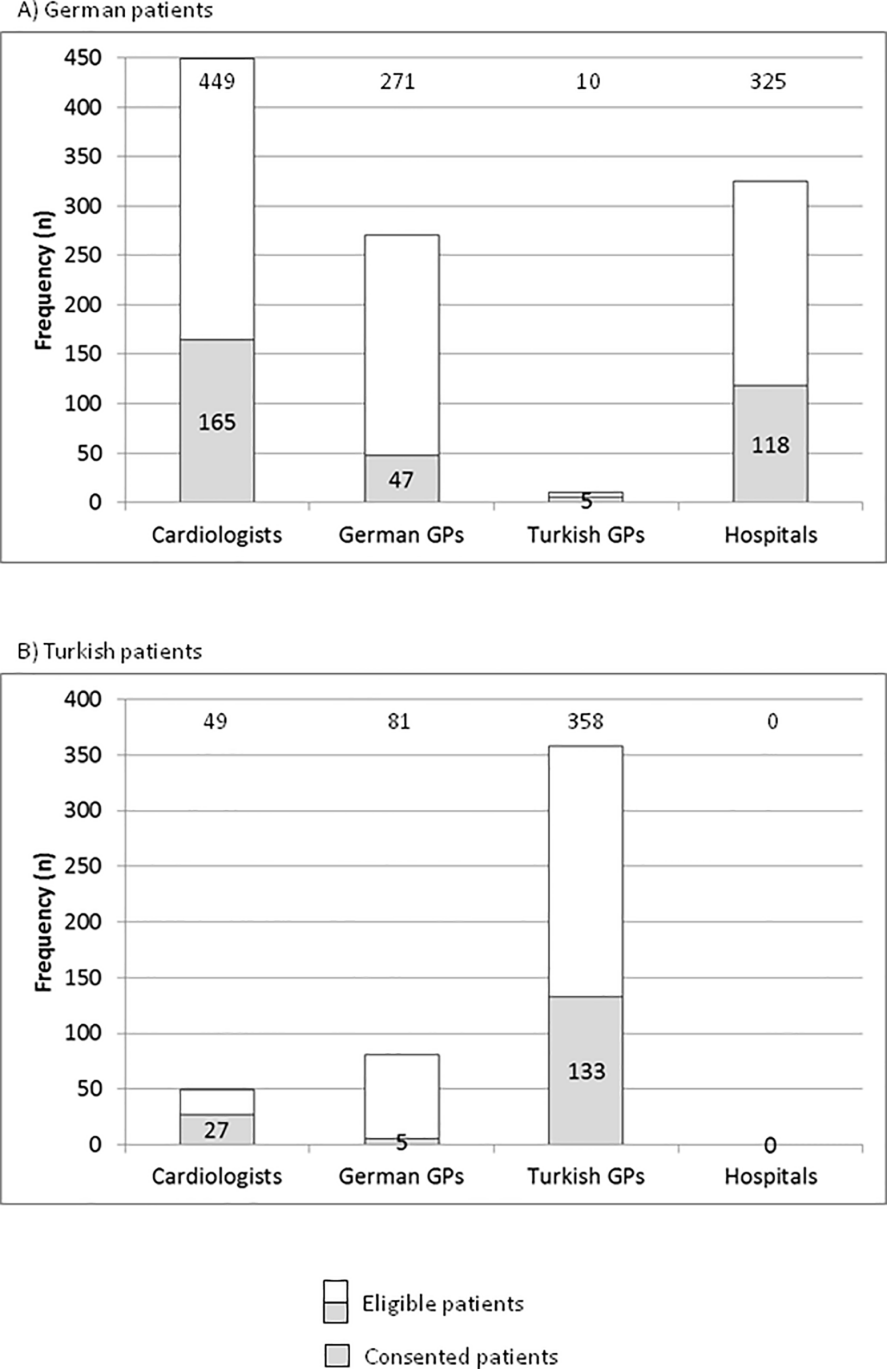
Frequency of German (A) and Turkish (B) eligible and consented patients by recruiting institutions.

The contribution of distinct participating health care sectors to the patient recruitment and inclusion differed significantly between German and Turkish patients (p<0.001, Fig 3). German patients were recruited and included predominantly by ambulatory cardiologists (n = 165, 49%) and in hospital (n = 118, 35%) and Turkish patients were recruited and included predominantly by GPs (n = 138, 84%) with 81% (n = 133) of Turkish patients recruited by Turkish GPs.

### Screening and consent rate

The screening modalities differed across institutions and partly were performed by practice assistants or physicians not transparent to the HNC staff. Hence, no results on the number of potentially eligible patients per institution and on the effectivity of screening can be provided.

After screening in hospitals and ambulatory practices 1,055 German and 488 Turkish patients were eligible for participation in the programme after final approval by the treating physician, with 325 German and no Turkish patients eligible from hospitals and 730 German and 488 Turkish patients eligible from ambulatory practices. Finally, 335 (32%) of the German patients and 165 (34%) of the Turkish patients consented for participation (p = 0.46 for comparison).

### Contacting and consent rate

In hospitals 121 (37%) of the 325 eligible German patients were directly contacted during the stay and 204 (63%) were contacted by mail letter after discharge of whom 88 (43%) responded. In ambulatory practices the majority of eligible Turkish and German patients were primarily contacted through telephone call (Table 1). The contact attempts by either mail letter or telephone call were significantly more successful in German (60%) compared to Turkish patients (52%, p = 0.005). This was due to a higher rate of missing phone numbers and of invalid address data in the patient records, a higher rate of lack of reachability by phone and a higher rate of missing response to mail letters in Turkish compared to German patients (Table 2).

**Table 1:** Mode and success of contacting eligible Turkish and German patients in ambulatory practices.

	Turkish	German	p-value
**Eligible patients, N**	488	730	
**First contact**			<0.001
**during clinics by doctor N(%)**	21 (4%)*	42 (6%)*	
**by telephone by HNC N(%)**	467 (96%)*	523 (72%)*	
**by mail by HNC N(%)**	-	165 (22%)*	
**Successful first contact**			
**by telephone by HNC N(%)**	184 (39%)	288 (55%)	<0.001
**by mail by HNC N(%)**	-	25 (15%)	-
**Secondary contact**			
**by mail by HNC N(%)**	283	230	
**by telephone by HNC N(%)**	-	-	
**Successful secondary contact**			
**by mail by HNC N(%)**	58 (20%)	102 (44%)	<0.001
**by telephone by HNC N(%)**	-	-	
**Overall successful contact**^**#**^	242 (52%)	415 (60%)	0.005

* (%) referring to eligible patients

^#^ referring to patients contacted through telephone or mail

**Table 2:** Causes for unsuccessful contacting eligible German and Turkish patients in ambulatory practices by telephone and mail.

	Turkish	German	p-value
**Missing phone number in records**	167/467 (36%)	129/523 (25%)	<0.001
**Not reachable by phone when number available**	116/300 (39%)	101/394 (26%)	<0.001
**Incorrect address data in records**	80/283 (28%)	29/395 (7%)	<0.001
**No response to mail letter when valid address available**	145/203 (71%)	239/366 (65%)	0.16

When referring consent rate to patients who could be successfully contacted, 50% (335/666) of German and 63% (165/263) of Turkish patients consented for participation (p<0.001). When restricting German patients to those recruited from ambulatory practices for better comparability results were virtually unchanged (consent rate referring to successfully contacted patients: 47%, p<0.001 for comparison with Turkish patients).

The inclusion of Turkish minority patients speaking little or no German into a nurse-led heart failure management programme is feasible subject to the condition that patients are approached by culture and language conform nurses and that specific minority characteristics are considered such as distinct health insurances. Compared to German patients Turkish patients showed a significantly higher willingness to initially participate in the programme but fewer Turkish patients were initially identified as eligible and the practical effort to enroll Turkish patients was higher due to barriers in contacting patients.

Patients with migration background comprise a substantial proportion of European populations which currently is further increasing and there is a particular need to improve integration of these people in the medical care system [16]. A major barrier of ethnic minority patients affecting access to health care is the linguistic limitation [17,18]. In Germany approximately 42% of Turkish immigrants speak less than moderate German [19]. We adapted staff and documents of our heart failure programme to Turkish language. Notably, our heart failure nurses also had Turkish migration background which might have been beneficial. Cultural conformity seems of high relevance for Turkish migrants [20] which was also observed in our study where 80% of our Turkish participants had a GP with Turkish migration background.

Despite language and cultural adaption we were not able to identify and enroll the aspired number of Turkish patients. Based on local demographics approximately 1,800 heart failure patients with Turkish immigration background could be expected in Cologne [21]. Only 488 Turkish patients were identified in our study although 71% of all ambulatory cardiologists in Cologne and several core Turkish GPs participated.

The question rises where potential barriers in the recruitment of Turkish heart failure patients could be found. One possibility is that Turkish patients might not find or actively deny access to the medical system overall. Our extensive efforts to increase awareness of the programme within the Turkish community did not improve recruitment rate suggesting that a lack of comprehensible information was not causative. Experiences from their mother country might affect readiness of Turkish patients such as for instance an adverse image of the GP in Turkey [22] who is an important gatekeeper of the health care system for migrants in particular [23]. Furthermore, medical services such as preventive interventions are rarely used by Turkish patients since a low significance is assigned to these interventions in Turkey [23]. Finally, other reasons such as education, the kind of family support or perception of advanced disease as fatalistic might also affect the readiness of Turkish minority patients to make demand of the health care system [24]. Of note, Turkish participants showed less advanced heart failure disease compared to Germans in our programme. This might suggest a restrictive attitude of Turkish patients with severe advanced heart failure or their socio-cultural ambience to engage advanced medical services.

Only a minority of the participating Turkish patients were recruited by specialized cardiologists. Given that most of the cardiologists in Cologne are successfully collaborating with HNC for many years and cardiological practices are distributed equally across Cologne also covering districts with high rate of immigrants, this might suggest that Turkish heart failure patients are less likely to be seen by cardiologists. This would have major implications since specialized treatment is an overall key quality parameter in heart failure care [15] and based on our experience with German patients cardiologists are motivated and effective in recruiting patients for heart failure programmes. Whether the presumably low appearance of Turkish patients in cardiologists`care might only be result of language restrictions and requirement of cultural conformity needs further study.

A major barrier in the recruitment of Turkish patients is contacting with missing or invalid data in 28 to 36%. Accordingly, only about half of eligible Turkish patients could be contacted successfully. This might be explained by cultural characteristics referring to the understanding of chronic disease and necessary continuous medical care. A survey on elderly Turkish migrants with diabetes showed that patients accepted diabetes as a disease and hence sought medical contact in very advanced stages only, when drug prescription was due or advanced medical interventions very necessary [25]. This “on-demand” comprehension of the medical health service in Turkish patients might contribute to the limited emphasize of valid and current contact data. Invalid address data and the low response rate in Turkish patients was reported in other health surveys [26] and might also be attributable to the fact that many elderly Turkish immigrants commute between Turkey and Germany [25].

Limitations of the study must be considered. The study was designed observational within the working routine of an established heart failure programme in order to get a pragmatic first insight into the feasibility of including Turkish minority patients. Thus, our results closely reflect status-quo of real-world care of a clearly defined and common population of Turkish heart failure patients speaking little or no German and being health insured. However, it was not aim of our study to answer emerging questions on mechanisms underlying the identified barriers which is crucial for the development of interventions to overcome these barriers since data assessment was limited and partly not systematic due to restrictions of participating physicians and patients. For instance, we did not further characterize the presumably heterogeneous group of Turkish minority patients with respect to time since immigration and sociocultural integration but also degree of familial support and psychosocial status which might differ from German patients and partly explain our findings independently of ethnicity. Notably, we did observe a significant difference regarding educational status between Turkish and German patients in a subgroup analysis of model eligible patients. Nonetheless, even if barriers in Turkish patients are not entirely result of ethnicity, this might not impact the relevance of our findings but might only affect targets of future interventions to improve accessibility of heart failure programmes for minority patients. Further, our results must be interpreted in the setting of the German health care system and patients with Turkish migration background. Issues such as open GP and health insurance selection by patients and reimbursement restriction of heart failure management programmes to distinct health insurance companies and regions might not apply to other systems. Individual barriers for programme inclusion might differ across ethnic and cultural minorities.

## Conclusions

In conclusion, the recruitment and inclusion of Turkish minority patients speaking little or no German into a heart failure management programme is feasible and patients initially show high consent rate. However, effort and costs are high due to inherent linguistic adaptions and barriers in identification and contacting of patients. Further study is needed to examine the barriers in identification of Turkish heart failure patients within or maybe outside the medical care system and to develop interventions to improve access of these patients to heart failure management programmes.

## Supporting information

S1 Table Actions taken to increase recruitment of Turkish heart failure patients. (DOCX)
