# Supplementary material for: Feasibility of including patients with migration background in a structured heart failure management programme: A prospective case-control study exemplarily on Turkish migrants
Source: PLoS One. 2017 Nov 8;12(11):e0187358. doi: 10.1371/journal.pone.0187358 (PMC5695597; doi:10.1371/journal.pone.0187358)
Supplement: S1 Table — (DOCX) [file pone.0187358.s001.docx]

| Informational material and presentations provided at Turkish embassy, Turkish cultural centers, Mosques, Ambulatory nursing service, Health insurance service points, Pharmacies  Health office |
| --- |
| Disposition of BNP point-of-care assays at 2 Turkish GP practices |
| Integration of another cardiological hospital department |
| Opening to patients with other health insurance companies |
